# Impact of Egg Components on Model Pasta Dough Rheology, Microstructure, and Mechanical Dough Rolling Energy

**DOI:** 10.17113/ftb.64.02.26.9220

**Published:** 2026-06-15

**Authors:** Berkay Berk, Şelale Öncü Glaue, Sevcan Ünlütürk

**Affiliations:** 1 Department of Food Engineering, Faculty of Engineering, Izmir Institute of Technology, İzmir, Türkiye; 2 Food Processing Department, Efes Vocational School, Dokuz Eylül University, İzmir, Türkiye

**Keywords:** egg, pasta dough, linear rheology, nonlinear rheology, extensional rheology, rolling

## Abstract

**Research background:**

Pasta is a widely consumed food usually made from durum wheat semolina. During production of pasta, dough kneading is the first step and varying degrees of deformation are applied to the dough. These deformations are related to the rheological properties of the pasta dough, which are affected by its ingredients.

**Experimental approach:**

In this study, three types of model pasta dough were prepared using water, whole eggs or egg yolks. To evaluate their properties, linear, nonlinear by large amplitude oscillatory shear (LAOS) and extensional rheological tests were conducted. The energy required during dough rolling was linked to the rheological properties of the dough samples.

**Results and conclusions:**

The results showed that the energy required during rolling is strongly correlated with crossover strain, oscillatory viscoelastic moduli, creep recovery compliances and extensional consistency coefficient. The addition of whole egg and egg yolk increased the rigidity of the model dough (plain: 6.32 kPa, whole egg: 28.48 kPa and yolk: 117.05 kPa Young’s modulus). The work done during rolling increased with the addition of egg (plain: 15.19 J, whole egg: 27.14 J and yolk: 33.02 J). After rolling, samples were cut, cooked, and their microstructure was evaluated. The effect of the addition of an egg on the microstructure of samples was observed as a lipid-coated, smoother surface and strong formation of a protein network.

**Novelty and scientific contribution:**

This study provides a comprehensive rheological characterisation of model pasta dough formulated with a whole egg or egg yolk using linear, nonlinear, and extensional tests. By linking these rheological properties to the mechanical energy required during rolling, the study introduces a novel approach to quantitatively predict processing behaviour from fundamental dough mechanics. The findings also highlight how egg yolk significantly enhances dough rigidity and alters microstructure, contributing to improved understanding of ingredient-function relationships in pasta formulation.

## INTRODUCTION

Cereals and their products are major staples in global diets. Pasta is a type of cereal product. Pasta dough is prepared by kneading durum wheat (*Triticum durum*) semolina or flour with water. Once the durum flour reaches the required moisture amount, it is added to a mixer and homogenised under high pressure. The homogenised dough is then kneaded again under vacuum to remove air bubbles and extruded into the desired shape before drying.

After hydrating the durum flour, the kneading and shaping stages take place and the rheology of the dough significantly influences process parameters such as rolling force, torque and energy consumption. During mixing and shaping, the dough undergoes deformation up to 500 % (*1*). Significant deformation aids dough development by breaking and reforming physical bonds. Proper dough development influences the shaping and subsequent drying processes. Dough preparation conditions and the ingredients used also have a significant impact on the physical properties of the dough.

Numerous studies have shown that various agents and their amounts added to the dough significantly affect dough rheology. For example, Li *et al.* (*2*) analysed the rheological properties of pasta dough, gluten gel, and gels made from different gluten fractions using a stress relaxation test. The study demonstrated how the gluten fractions of wheat affected the rheological properties of the dough. Larrosa *et al.* (*3*) made gluten-free pasta dough from corn flour and starch using egg and obtained desirable dough consistency through the binding effect of the egg.

During rolling of the pasta dough, deformations caused by biaxial extensional strain were observed. Several studies discuss the effect of ingredients on the biaxial extensional properties of the dough. Dufour *et al.* (*4*) discuss extensional rheological properties as affected by water content of the dough and mixing time. The findings revealed that both water and mixing affected the gluten network structure by leveraging the protein strands, resulting in increased extensional consistency. In another study conducted by Yang *et al.* (*5*), variations in deformation by different methods of dough kneading were investigated using extensional rheology. They found that the best gluten alignment was achieved by single direction pressing and bidirectional rolling due to the arrangement of the protein strands subjected to external force.

In this study, three different pasta dough samples were prepared separately using water, a whole egg, or egg yolk. The rheological properties of the dough were analysed and related to the technological properties of pasta dough rolling process. To achieve this, the study investigated the following: (*i*) linear viscoelastic properties, (*ii*) nonlinear viscoelastic properties, (*iii*) extensional rheological properties of the pasta dough, (*iv*) parameters of the dough rolling process, and (*v*) quality characteristics of the finished pasta.

## MATERIALS AND METHODS

### Materials

The flour used in this study was made from *Triticum durum* (Tellioğlu Gıda, Balıkesir, Türkiye), containing in g per 100 g of flour: fat 0.7, carbohydrate 71, protein 12.5, ash 0.62 and moisture 15.18. The mean particle size of the flour was (83.0±1.9) µm. To prepare the dough samples, distilled water, whole egg and egg yolk were used separately. The eggs were purchased from a local market in İzmir, Türkiye. The moisture mass fraction of the egg was determined to be (49.6±2.1) % for egg yolk and (90.5±1.9) % for egg albumen. The yolk-to-albumen ratio of the eggs was measured as (43.6±3.2) %. The soluble protein content of the egg and egg yolk was determined according to the Bradford method (*6*). The results indicated that the protein mass fraction on dry egg yolk basis was (0.23±0.03) g/g, and on dry egg albumen basis (0.79±0.09) g/g. The lipid mass fraction of egg yolk was measured according to the method of Lin *et al.* (*7*) and found to be (36.2±1.2) % .

### Model pasta dough preparation

In this study, three different dough samples were prepared separately using water, whole egg, or egg yolk. In all formulations, 100 g of flour was mixed with 50 g of liquid, either water, whole egg, or egg yolk. The amount of liquid was kept to a minimum, considering the operating conditions of the equipment used. The dough was kneaded using a food mixer (MUM5; Bosch, Gerlingen, Baden-Württemberg, Germany) with a kneading hook (Bosch) at speed 4 (60 rpm) for 5 min.

### Rheological analyses

All rheological measurements were performed immediately after kneading the dough using a DHR-20 rheometer (TA Instruments, New Castle, DE, USA) with a 50 mm parallel plate equipped with a Peltier system. The measurements were conducted at 25 °C, and the lateral edge of the dough was covered with paraffin oil to prevent moisture loss during the experiment.

#### Strain sweep test

To determine the linear (LVR) and nonlinear viscoelastic regions, a strain sweep test was performed. Strain was swept from 0.01 to 100 % at an oscillatory frequency of 10 rad/s. The measurement gap was set to 2 mm, and the axial force was allowed to relax below 10 N prior to measurement.

#### Frequency sweep test

In LVR, a frequency sweep test was conducted at 0.02 % strain between 0.1 and 10 rad/s. The measurement gap was set to 2 mm, and the axial force was allowed to relax below 10 N before measurement. The storage (*G*') and loss (*G*") moduli and complex viscosity (η*) were modelled using power law models as given in the following equations (*8*):


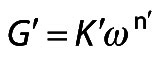
 /1/


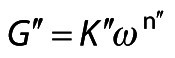
 /2/

where *K*' and *K*" are consistency indices (Pa·s^n’^ and Pa·s^n’’^, respectively), *n*' and *n*" are oscillatory flow behaviour indices of storage and loss moduli, respectively and *ω* is angular frequency.

#### Creep recovery test

The creep recovery test was conducted under a constant stress (*σ*_0_) of 200 Pa during the creep period and 0 Pa during the recovery period, with a gap height of 2 mm. The durations of the creep and recovery periods were 120 and 180 s, respectively. The strain data were then converted to creep compliance (*J*_c_(*t*)) and recovery compliance (*J*_r_(*t*)).

J_c_(*t*) data were modelled with the 6-element Burgers model (comprising one Maxwell and two Kelvin-Voigt elements in series) as shown in the following equation (*8*):


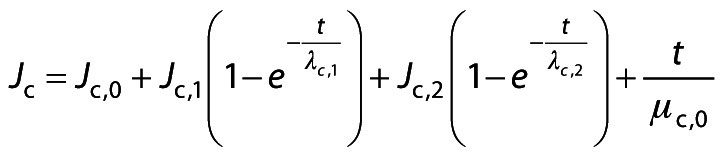
 /3/

where *J*_c,0_ is the instantaneous compliance (Pa^-1^), *J*_c,1_ is the first element retarded compliance (Pa^-1^), *λ*_c,1_ is the first element retardation time (s), *J*_c,2_ is the second element retarded compliance (Pa^-1^), *λ*_c,2_ is the second element retardation time (s) and *μ*_c,0_ is the zero shear viscosity (Pa·s) of the free dashpot.

*J*_r_(*t*) data were modelled with the 6-element Burgers model (one Maxwell and two Kelvin-Voigt elements in series) in the following equation (*8*):


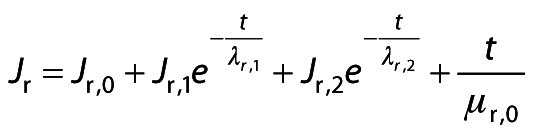
 /4/

where *J*_r,0_ is the instantaneous recovery compliance (Pa^-1^), *J*_r,1_ is the first element retarded recovery compliance (Pa^-1^), *λ*_r,1_ is the first element recovery retardation time (s), *J*_r,2_ is the second element retarded recovery compliance (Pa^-1^), *λ*_r,2_ is the second element recovery retardation time (s) and *μ*_r,0_ is the zero shear viscosity (Pa·s) of the free dashpot.

#### Large amplitude oscillatory shear analysis

Large amplitude oscillatory shear (LAOS) properties of the dough samples were measured outside the LVR (*9*). The data collected were processed using MITlaos software (*10*). From these data, nonlinear measures (*G*’_L_, *η*’_L_, *S* and *T*) and Lissajous-Bowditch curves obtained.

#### Extensional rheological analysis

Biaxial extensional rheology measurements were conducted in the compression mode of the rheometer at a compression speed of 1.667·10^-5^ m/s, with a sample diameter of 25 mm and a height of 10 mm. A constant volume approach was used, and the upper and lower plates were lubricated with paraffin oil. Biaxial extensional stress *vs* extensional strain rate data were obtained. Young’s modulus (YM) was calculated in the LVR using the slope of the stress-strain curve. Biaxial extensional viscosity *vs* extensional strain rate data were fitted to the power law model using the following equation (*8*):


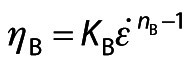
 /5/

where *η*_B_ is biaxial extensional viscosity (Pa·s), *K*_B_ is the power law constant (Pa·s^nB^) and *n*_B_ is extensional thinning index. Maximum extensional strain rate (*ε̇*_b,max_) was kept low (0.24 s^-1^) to prevent lubricant loss.

### Dough rolling process analysis

The kneaded dough samples were hand rolled to 5 mm thickness before machine rolling, then cut into 10 cm×10 cm squares. A digital ammeter (DT-9987; CEM, Shenzhen, PR China) was serially connected to pasta rolling machine (*d*_roller_=2.5 cm) (Atlas 150; Marcato, Campodarsego, Italy). While rolling with a roller gap of 2 mm (no. 3), current measurements were taken in AC mode and time *vs* current data were obtained. The roller speed and the operating voltage were recorded as 60 rpm and 230 V, respectively. The angular impulse is defined as the area under the torque *vs* time curve (*11*). The work done by the rollers can be calculated using the following equation (*11*):


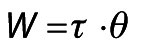
 /6/

where *W* is the work done (J), *τ* is the torque (N·m) and *θ* is angular displacement in rad. When the rotational displacement is multiplied by *τ*, the total work done to roll the dough is obtained.

### Microstructure of the raw and cooked pasta

Cut dough samples were dried before and after cooking in a laboratory oven (Incudigit-36L; JP Selecta, Abrera, Spain) at 50 °C and 15 % relative humidity for 24 h. Convection mode was not used to prevent rapid drying and surface cracks. Then, 10 g of each dried sample were cooked in 100 mL boiling distilled water for 5 min. The cross-sectional morphologies of dried pasta dough before and after cooking were examined using a scanning electron microscope (SEM, 250 Quanta FEG; FEI Company, Hillsboro, OR, USA) at magnification rates of 500 and 2500×. Samples were gold-coated with a sputter coater (Emitech K550X; Quorum Technologies Ltd., Laughton, UK) under 10 mA for 30 s.

### Statistical analysis

All formulations were prepared twice, analyses were conducted in duplicate and differences among the samples were tested using analysis of variance (ANOVA) with Tukey’s comparison test at the 95 % confidence level. The relationship between rheological properties and energy required for dough rolling was investigated using the Pearson correlation test at the 95 % confidence level. The analyses were conducted using Minitab statistical software (*12*).

## RESULTS AND DISCUSSION

### Rheological analyses of pasta dough

#### Linear viscoelastic properties

**Fig. 1:**
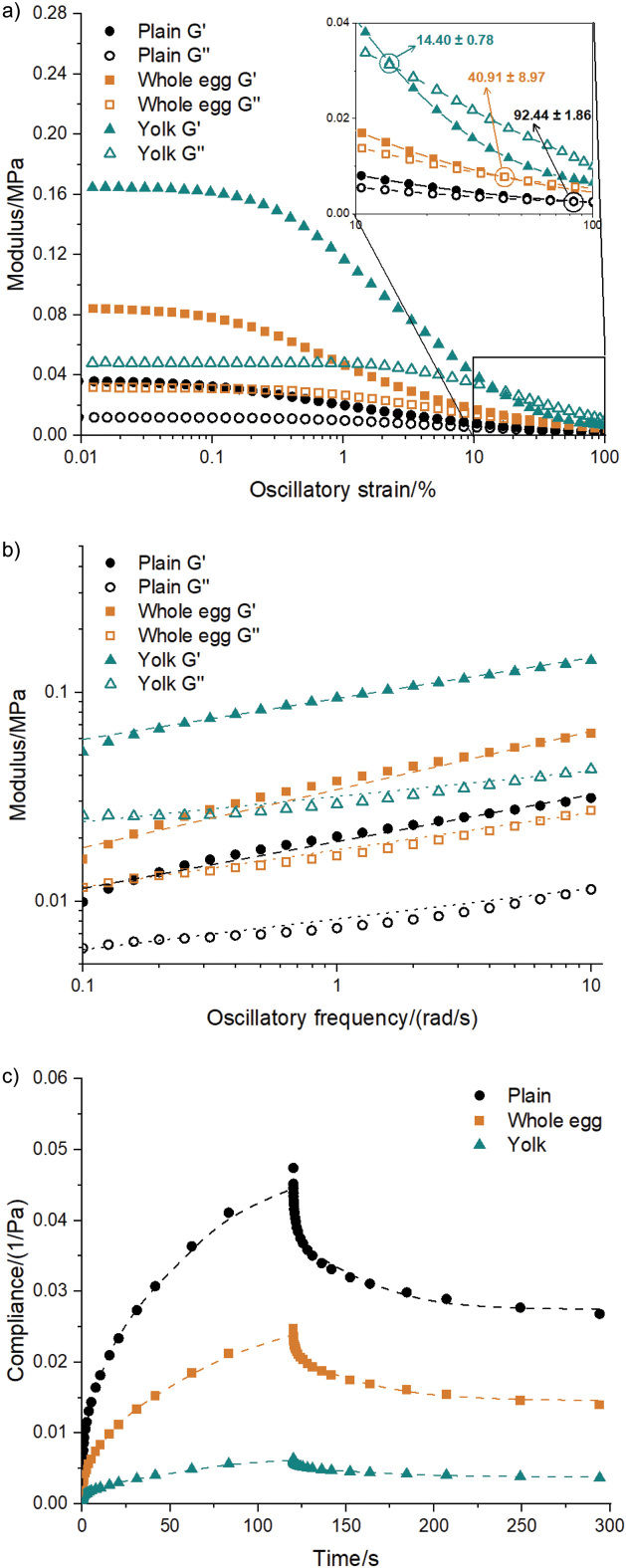
The graphs show: a) strain sweep (with crossover strain numerical values marked in the inserted graph), b) frequency sweep and c) creep recovery profiles of the dough samples prepared with only water (plain, circles), whole egg (squares), and egg yolk (triangles)

Fig. 1 shows the linear viscoelastic rheograms. A strain sweep test was conducted to determine the linear viscoelastic region, and the strain sweep profiles of pasta doughs are shown in Fig. 1a.

Airey *et al.* (*13*) identified the nonlinear viscoelastic region of the strain sweep test data by considering the point where 5 % of the initial modulus decayed. The results of this study are consistent with previous research, showing that all samples exhibited nonlinear behaviour after a strain of 0.05 %. The strain values at which the dough samples entered the nonlinear viscoelastic region were recorded as 0.05 % for the plain sample, 0.08 % for the whole egg sample, and 0.20 % for the egg yolk sample. Similarly, Mastromatteo *et al.* (*14*) prepared bread dough using durum wheat and found that the linear viscoelastic region (LVR) was between 0.02 and 0.065 %, consistent with the plain samples. The highest moduli were observed in the egg yolk sample. Since the dough is characterised as a viscoelastic solid, the storage modulus (*G*') of each sample was greater than the loss modulus (*G*"). Within the LVR, the difference between *G*' and *G*" was more pronounced than in the nonlinear region, indicating that the elastic behaviour dominates until the structure starts to break down. As the strain amplitude increased, both moduli decreased, reflecting the disruption of the gluten-starch network. Similar behaviour was reported by Yazar *et al.* (*1*), who attributed this decrease to overstretching of the gluten network and the consequent loss of its structural integrity. Erturk *et al.* (*15*) further explained that, after the gluten network is overstretched, starch-starch interactions become more dominant, governing the viscoelastic response at large strain amplitudes. In this study, the observed reduction in *G*' and *G*" with increasing strain can therefore be explained by the transition from a gluten-dominated to a starch dominated structural response.

Within the measurement range, crossover strains were observed (see Fig. 1a). At higher crossover strain value, viscous behaviour dominates, and the dough behaves in a liquid-like manner. The crossover strain values are given in Table 1. The highest crossover strain was observed in the plain sample. The addition of whole egg or egg yolk prevents gluten network formation due to lipids in the egg yolk. In the study by Alamprese *et al.* (*16*), the break load of raw pasta dough decreased as the yolk amount increased, which they explained as the weakening of the protein network due to inhibition by egg yolk lipids. The reason for the decreasing crossover strain with increasing egg yolk content could be the same.

**Table 1:** Crossover strain values and the rheological model fitting parameters

		**Sample**		
**Test**	Parameter	Plain	Whole egg	Yolk
**Strain sweep**	Crossover strain/%	(92.4±1.9)^A^	(40.9±9.0)^B^	(14.4±0.8)^C^
**Frequency sweep**	*K*'/(MPa·s^n’^)	(0.020±0.001)^B^ R^2^=0.98	(0.036±0.006)^B^ R^2^=0.99	(0.092±0.006)^A^ R^2^=0.99
	*n'*	(0.226±0.003)^B^ R^2^=0.98	(0.277±0.001)^A^ R^2^=0.99	(0.204±0.002)^C^ R^2^=0.99
	*K*"/(MPa·s^n’’^)	(0.008±0.000)^C^ R^2^=0.96	(0.017±0.003)^B^ R^2^=0.98	(0.031±0.002)^A^ R^2^=0.94
	*n"*	(0.130±0.001)^B^ R^2^=0.96	(0.18±0.02)^A^ R^2^=0.98	(0.116±0.002)^B^ R^2^=0.94
**Creep compliance**	*J*_c0_/(1/Pa)	(0.0026±0.0001)^A^ R^2^=0.99	(0.0014±0.0004)^B^ R^2^=0.99	(0.0004±0.0001)^C^ R^2^=0.99
	*J*_c1_/(1/Pa)	(0.0061±0.0004)^A^ R^2^=0.99	(0.0023±0.0002)^B^ R^2^=0.99	(0.0006±0.0001)^C^ R^2^=0.99
	*J*_c2_/(1/Pa)	(0.012±0.002)^A^ R^2^=0.99	(0.0069±0.0003)^B^ R^2^=0.99	(0.0016±0.0004)^C^ R^2^=0.99
	*λ*_c1_/s	(0.320±0.108)^A^ R^2^=0.99	(0.5±0.1)^A^ R^2^=0.99	(0.16±0.06)^A^ R^2^=0.99
	*λ*_c2_/s	(13.0±0.1)^B^ R^2^=0.99	(16.1±0.2)^A^ R^2^=0.99	(7.4±0.7)^C^ R^2^=0.99
	*µ*_c0_/(Pa·s)	(4069±393)^B^ R^2^=0.99	(8518±804)^B^ R^2^=0.99	(26355±1678)^A^ R^2^=0.99
**Recovery compliance**	*J*_r0_/(1/Pa)	(0.0086±0.0007)^A^ R^2^=0.98	(0.011±0.002)^A^ R^2^=0.99	(0.0014±0.0002)^B^ R^2^=0.98
	*J*_r1_/(1/Pa)	(0.0072±0.0003)^A^ R^2^=0.98	(0.0036±0.0008)^B^ R^2^=0.99	(0.0009±0.0001)^C^ R^2^=0.98
	*J*_r2_/(1/Pa)	(0.030±0.001)^A^ R^2^=0.98	(0.0082±0.0004)^B^ R^2^=0.99	(0.0035±0.0008)^C^ R^2^=0.98
	*λ*_r1_/s	(0.010±0.004)^B^ R^2^=0.98	(1.1±0.1)^A^ R^2^=0.99	(1.07±0.09)^A^ R^2^=0.98
	*λ*_r2_/s	(97.5±6.8)^B^ R^2^=0.98	(47.7±9.1)^C^ R^2^=0.99	(127.4±4.0)^A^ R^2^=0.98
	*µ*_r0_/(Pa·s)	(23436±16662)^B^ R^2^=0.98	(141327±14655)^A^ R^2^=0.99	(119029±12203)^A^ R^2^=0.98
**Extensional rheology**	*K*_B_/(MPa·s^nB^)	(0.035±0.004)^B^ R^2^=0.96	(0.047±0.000)^B^ R^2^=0.99	(0.10±0.01)^A^ R^2^=0.97
	*n*_B_	(0.389±0.007)^B^ R^2^=0.96	(0.29±0.01)^C^ R^2^=0.99	(0.412±0.009)^A^ R^2^=0.97

Mean values with different letters in superscript within a row are significantly different. R^2^ represents the adjusted R^2^ of the fitted model

The frequency sweep profile of the samples is shown in Fig. 1b. In the LVR, storage modulus is greater than the loss modulus for all samples. The data obtained were fitted to the power law and the fitting results are given in Table 1.

For both *G*' and *G*", all oscillatory flow behaviour indices (*n*) are less than 1, indicating that all samples had shear thinning behaviour. Similarly, in the study by de la Peña *et al.* (*17*), the pasta dough prepared with various moisture contents (30–34 %) showed shear thinning behaviour. Both *n*' and *n*" were highest in samples made with whole eggs, indicating that increased oscillatory frequency results in a thicker dough than in the other samples. For *n*', the frequency dependency decreases in the following order: whole egg, plain, and yolk samples. In contrast, for *n*", the plain and yolk samples exhibit similar frequency dependencies. In this study, the final dough moisture mass fractions ranged approx. from 48 to 56 %, which are considerably higher than those obtained by de la Peña *et al.* (*17*). Such higher hydration degree could be used for model pasta dough. The addition of whole egg and egg yolk leads to enhanced protein network plasticisation and more pronounced viscoelastic responses. While de la Peña *et al.* (*17*) observed a decrease in apparent viscosity with increasing hydration, a similar trend can be observed in the present study, as higher water content facilitates molecular mobility. This reduces the dough resistance and results in shear thinning behaviour. The yolk sample exhibited the highest *K*', indicating the highest stress response to oscillatory shearing. The higher *K*' value of the sample containing egg yolk may be related to the fact that the egg yolk is more viscous and has a higher solid content than the samples containing whole eggs and water. Even though the presence of lipids interrupts the gluten network formation and weakens the dough structure, the higher consistency coefficient at LVR could be attributed to the limited water mobility due to more viscous nature of the egg yolk. Similar trend was observed in the *K*" values, which are related to the viscous properties of the sample. Since the dough is characterised as a viscoelastic solid and all samples have greater *K*' than *K*", the increasing *K*" values were observed as the viscosity of the liquid ingredient increased. From the perspective of dough processing, the frequency dependency of the samples is a crucial property for dough stability (*8*, *18*). Higher frequency dependency results in more unstable dough, which could be a problem during mechanical processes. After kneading, rolling and cutting steps follow, and the instability such as disintegration or spring-back of the dough during these mechanical processes could interrupt proper handling of the overall process steps.

The results of the creep recovery test are shown in Fig. 1c. *J*_c_(t) and *J*_r_(t) data fitted to Burgers model (R^2^_adj_>0.98) are given in Table 1. The six-element Burgers model was found to describe both the creep and recovery compliance data and it provides valuable insights into viscoelastic materials. Due to the second curvature of both the creep and recovery compliance data, an additional Kelvin-Voigt element in series with the four-element Burgers model was needed to describe the compliance behaviour. In the study by Van Bockstaele *et al.* (*19*), different wheat flour cultivars were used to prepare bread dough, and the creep recovery behaviour was investigated. Similar to our findings, they discovered that the best model for describing the creep behaviour was the six-element Burgers model, while the recovery behaviour was well modelled by the five-element Burgers model (the six-element Burgers model without the zero shear viscosity).

In the creep phase, the first and second element compliances of the plain sample were significantly greater than those of the other samples. Given the lower consistency index of the plain sample, this difference can be attributed to its greater ability to deform under shearing. Zhang *et al.* (*20*) studied the effect of freeze-thaw cycles on the rheological properties of pasta dough and showed that an increasing number of cycles reduced the rigidity of the dough, which was explained by the lowered G'. As rigidity increased, creep compliance decreased, which aligns with the findings of this study. The retardation times of the individual Kelvin-Voigt elements during creeping consisted of two distinct values, representing shorter and longer retardation times. *λ*_c1_, which denotes the shorter retardation time, showed no statistically significant difference between the samples (p<0.05). For the longer retardation times in the creep phase, the highest value was observed in the whole egg sample. The increased protein content in the whole egg sample may contribute to the longer retardation time, likely due to the formation of a stronger network during kneading. The zero shear viscosity of the yolk sample was the highest and this value correlated with the consistency index obtained in the frequency sweep test.

During the recovery phase, the highest instantaneous compliance values were observed in the plain and whole egg samples. As with creep compliance, this phenomenon could be explained by the rigidity of the dough. For a shorter retardation time, the plain sample exhibited the lowest value, indicating that it recovered from the deformation applied during the creep phase more quickly. The longest retardation time was observed in the yolk sample, followed by the plain and then the whole egg samples. This could be attributed to the strength of the dough. After the rapid rearrangement of the macromolecules in the network associated with the short retardation time, the recovery from deformation took longer in the yolk sample, which was characterised by its stability. The zero shear viscosity during the recovery phase was the lowest in the plain sample.

#### Nonlinear viscoelastic properties

**Fig. 2:**
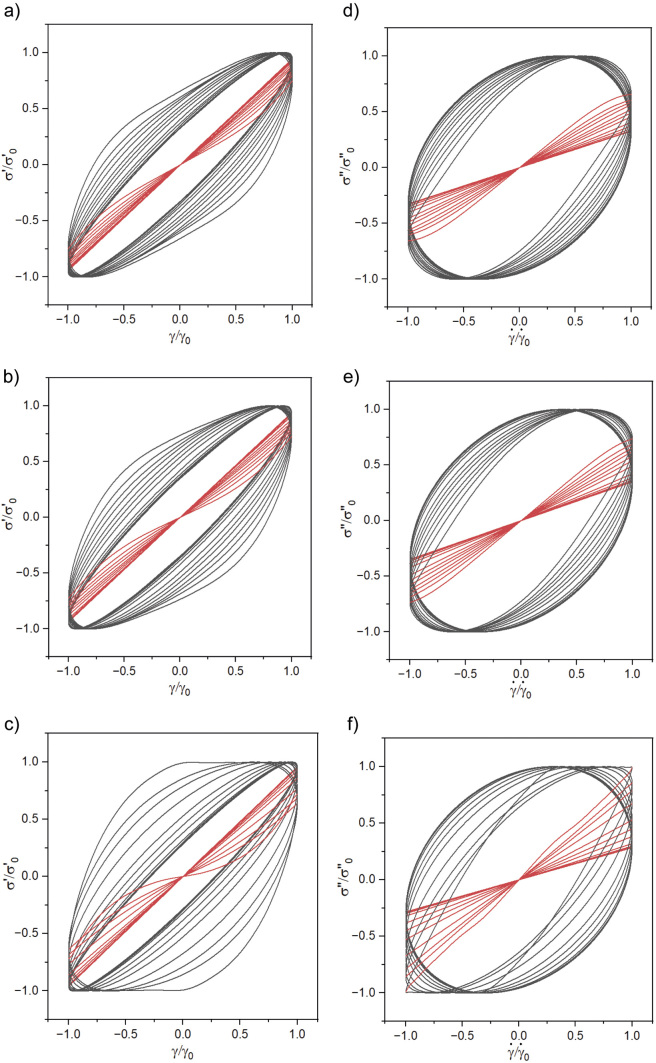
Lissajous-Bowditch curves of elastic components of: a) plain, b) whole egg, c) yolk and viscous components of: d) plain, e) whole egg, and f) yolk samples

Lissajous-Bowditch curves for both the elastic and viscous components of the samples are shown in Fig. 2. All samples in the Lissajous-Bowditch curves had clockwise rotation, indicating intracycle strain stiffening behaviour (*21*). Using Lissajous-Bowditch curves, both qualitative and quantitative measures can be obtained. For quantitative comparison, the shape of the curves is informative. A circular structure indicates a viscous response, while a narrow ellipse indicates an elastic response of the stress to the strain input (*22*). In the LVR, the stress response to strain was linear, but outside the LVR, the Lissajous-Bowditch curves were distorted. In the gluten- and starch-rich formulations, undistorted Lissajous-Bowditch curves were observed due to the reorientation of starch without disruption of the gluten network (*1*). As a result, elasticity was provided by starch, and intracycle linearity was observed. In the quantitative analysis of Lissajous-Bowditch curves, the area enclosed by the elastic component curve serves as a measure of energy storage in the network against deformation. In this study, it was found that the yolk sample had a greater enclosed area as the strain increased, which means that higher shearing was required to deform this material. The trends in the increase of the enclosed areas for the plain and whole egg samples were similar. Energy dissipation during a cycle was discussed in the studies of Duvarci *et al.* (*21*) and Ozcan *et al.* (*23*), which explained the projection area of the stress-strain curves.

**Fig. 3:**
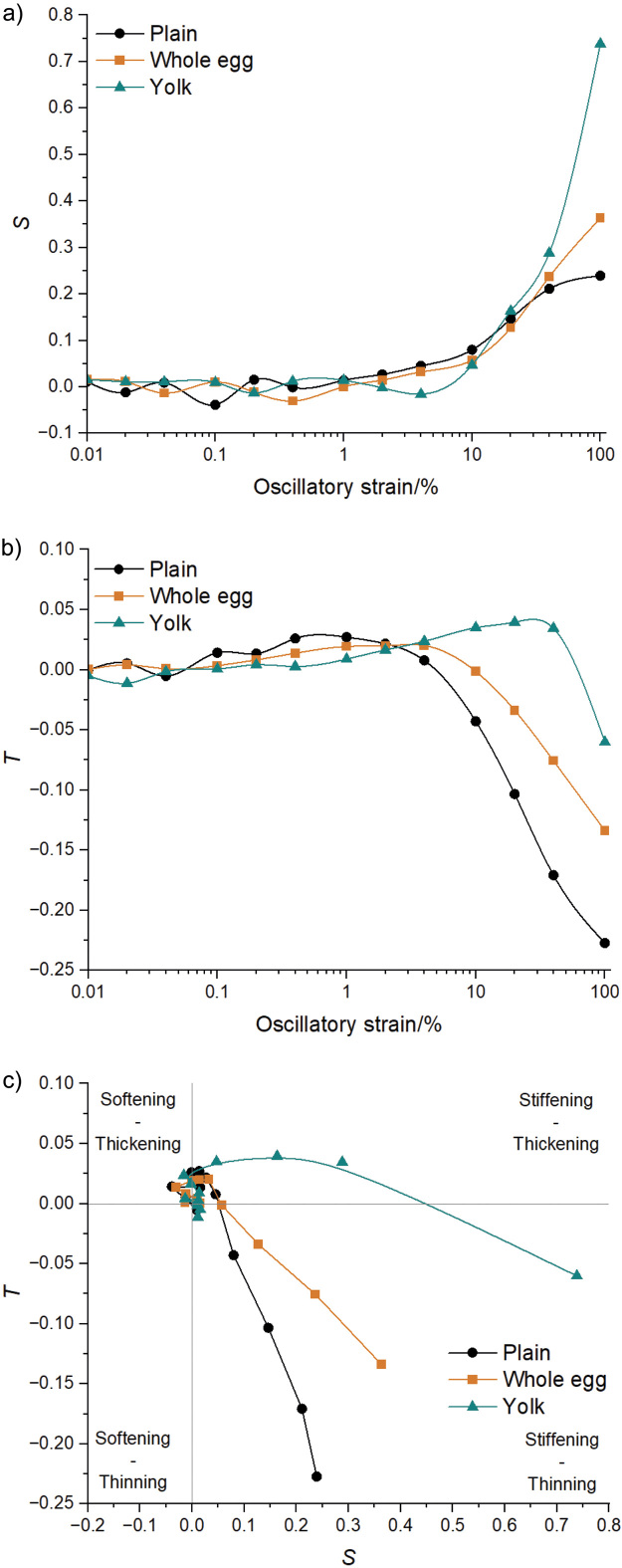
The ratios of: a) strain stiffening (*S*) and b) thickening (*T*) with respect to oscillatory strain amplitude; c) *S vs T* values

Strain stiffening ratio (*S*) and thickening ratio (*T*) are shown in Fig. 3a and Fig. 3b, respectively. The physical meaning of the *S* value is intracycle strain softening when less than 0 and intracycle strain stiffening when greater than 0 (*10*). The plain and whole egg samples showed strain stiffening behaviour after 1 % oscillatory strain, while the yolk sample exhibited this behaviour after 10 % strain. The *T* value indicates shear thinning when less than 0 and shear thickening when greater than 0. The plain sample had the lowest strain value at which dough passes to shear thinning behaviour. The next formulation was with whole egg, and the highest strain requirement was observed in the yolk sample. Simultaneous classification of stiffening and thickening behaviour based on *S* and *T* is shown in Fig. 3c. At small strain values, all samples exhibited shear thickening and strain softening. As the strain increased, all samples first entered the strain stiffening region and then exhibited shear thinning behaviour. Erturk *et al.* (*15*) explained this phenomenon as the gluten network disruption under higher shearing. It was speculated that the lipids in the yolk sample inhibited gluten network formation. This trend may be attributed to the rigidity of the yolk dough, which might cause it to exhibit shear thinning behaviour at higher deformation levels. This result provides important insight into the dough machinability, indicating that the samples containing egg yolk can resist deformation at a higher level compared to the other formulations.

#### Extensional rheology

**Fig. 4:**
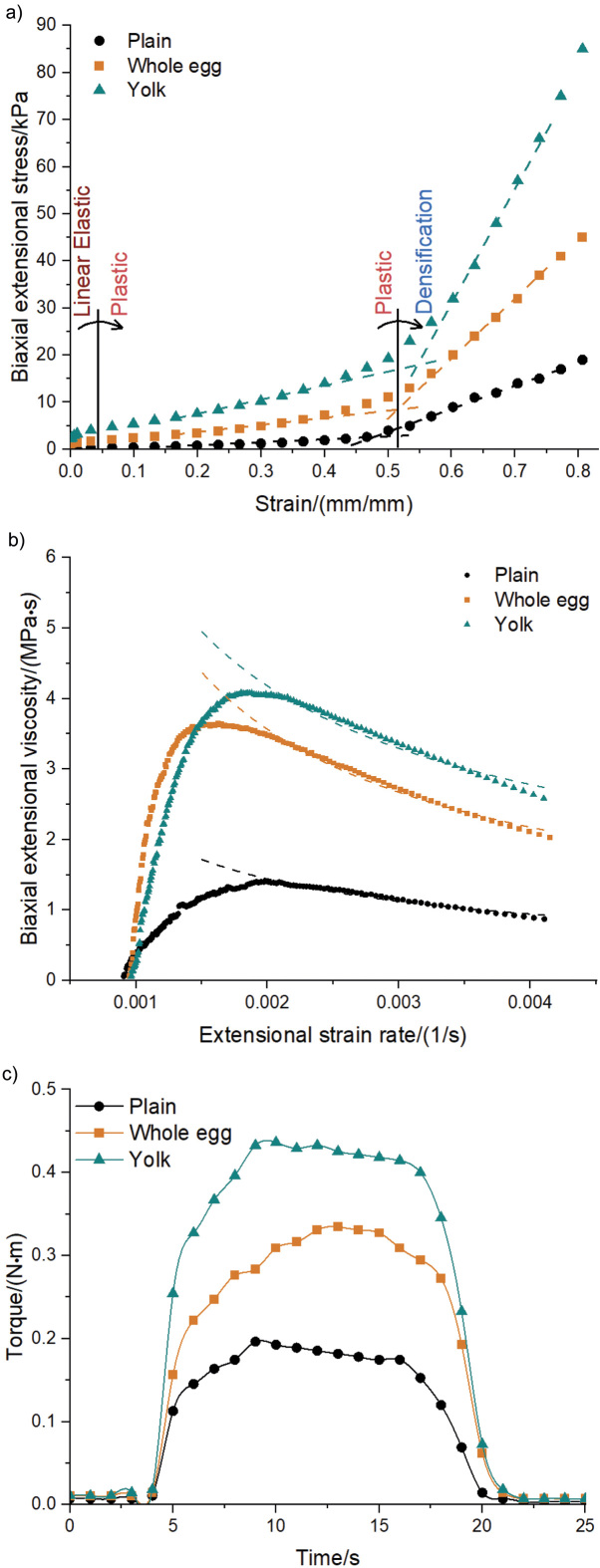
The graphs show: a) biaxial extensional stress *vs* strain, b) biaxial extensional viscosity profile and c) torque change during rolling

Biaxial extensional stress *vs* strain data for all samples are shown in Fig. 4a. It was found that the stress response to the biaxial compression was the highest in the yolk sample. The Young’s moduli (YM) of the samples were calculated as 6.32, 28.48 and 117.05 kPa for the plain, whole egg and yolk samples, respectively. The YM were calculated up to a strain of 0.01, where the material entered the nonlinear viscoelastic region. Yu and Ngadi (*24*) investigated the mechanical properties of noodle dough containing guar gum and varying moisture content. Their results showed that the decreasing moisture content increased the YM, which is in agreement with the findings of this study, and that the gum addition increased the YM by providing stronger interactions between water and macromolecules. The regions observed in the stress-strain curve were the linear elastic, plastic, and densification regions (*25*). The YM was calculated in the initial region, and as the material transitioned into the densification region, the stress difference between the samples increased. In this study, the highest densification was observed in the yolk sample. Higher strain values in dough machinability can lead to increased stress, which is directly proportional to the degree of dough rolling. This results in a higher energy requirement during rolling of the dough; in other words, the force applied to achieve the targeted strain is greater. On the other hand, the plain sample had the lowest densification, meaning that the strain induction during rolling did not cause excessive collapse in the polymeric network formed during kneading (*4*).

The biaxial extensional viscosity profile is shown in Fig. 4b. The viscosity profiles of the samples were fitted to the power law, and the model parameters are given in Table 1. In terms of *K*_B_, the plain and whole egg samples behaved similarly and were less consistent than the yolk sample. It was observed that the extensional thinning index decreased in the order of yolk, plain and whole egg samples. Liao *et al.* (*26*) studied the biaxial extensional viscosity profile of rolled noodle dough and explained the extensional thinning index as the orientation of the molecular chain of glutenin. The samples in this study also contained egg proteins, meaning that the orientation of the egg proteins also contributed to the thinning index. A lower thinning index indicates a greater ability to deform before structural breakdown. The whole egg sample had the lowest *η*_B_ (Table 1) value, meaning that its extensibility without structural damage was the most favourable for the rolling process.

### Dough rolling process

The electric current measured during the rolling process was determined by subtracting the current measured when no material was rolled from the total current during the entire rolling process. The current values were converted into torque data, as shown in Fig. 4c. The dough was fed into the roller section at the 4^th^ second, and deposition was observed at the 20^th^ second, indicating that the rolling process lasted 16 seconds. The calculated angular impulse for the plain, whole egg and yolk samples was (2.42±0.09), (4.32±0.05) and (5.26±0.09) N·m·s, respectively.

The estimated values for the work done by the rollers in the pasta roller machine were (15.2±0.6), (27.1±0.3) and (33.0±0.6) J for the plain, whole egg and yolk samples, respectively. In the study conducted by Mohammed *et al.* (*27*), the rolling process of potato-based dough was examined, with energy consumption for the rollers reported to be between 31 and 40 J/kg of dough. These values are higher than those observed in the present study. This difference may be due to the gluten content in pasta dough, which facilitates a more efficient rolling process.

### Correlation between rheological properties and the rolling process

**Fig. 5:**
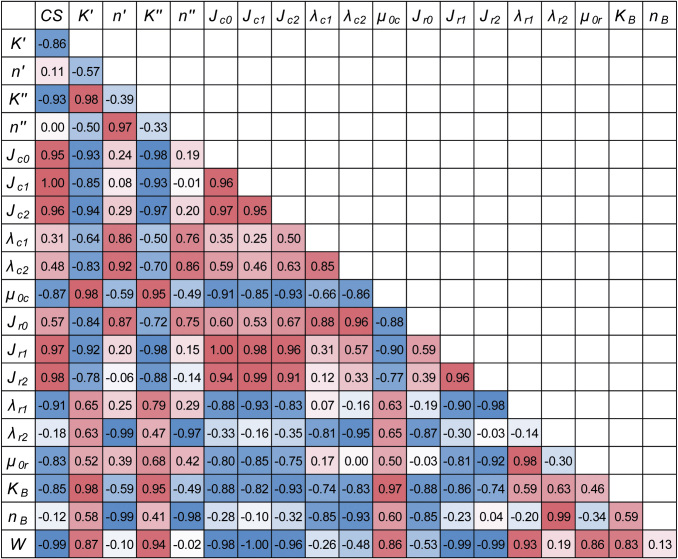
Pearson’s correlation matrix of the parameters. Numbers in the coloured boxes represent the corresponding correlation coefficients. The colour scale is set so that red indicates positive correlation and blue indicates negative correlation

Pearson’s correlation test with a 95 % confidence level was conducted to determine the interactions between the rheological properties and the evaluated dough rolling process outputs. The correlation matrix is shown in Fig. 5.

The correlations between rheological properties and the work done (*W*) by the roller during the rolling process in the pasta machine were examined. The models developed for the significant correlations are presented in Table 2.

**Table 2:** Models developed for the correlation analysis with the work done (*W*)

**Correlated parameter**	**Model**	**R^2^**
**Crossover strain (CS)**	=-4.3509∙*W*+158.5	0.98
***K'***	=0.0037∙*W*+0.0427	0.76
***K"***	=0.0012∙*W* -0.00116	0.89
***J*_c,0_**	=-0.0001∙*W* +0.0044	0.96
***J*_c,1_**	=-0.0003∙*W* +0.0107	0.99
***J_c,2_***	=-0.0006∙*W* +0.0209	0.92
***μ*_c,0_**	=1120.9∙*W* -15167	0.74
***J*_r,1_**	=-0.0003∙*W* +0.0126	0.98
***J*_r,2_**	=-0.0016∙*W* +0.0533	0.97
***λ*_r,1_**	=0.0639∙*W* -0.8827	0.87
***μ*_r,0_**	=6009.6∙*W* -56311	0.73
***K*_B_**	=0.0032∙*W* -0.0203	0.70

R^2^ represents the adjusted R^2^ of the model fitted

A strong negative correlation was found for the crossover strain value, indicating that a higher crossover strain requires less work to roll the dough. This may be due to rolling occurring within the viscoelastic region. If the dough with a higher crossover strain is rolled in the LVR, the structure can stretch and align evenly, which may require less energy to process. In contrast, a lower crossover strain leads to a rapid pass to the nonlinear viscoelastic region, where structural breakdown results in uneven orientation of the macromolecules. This may increase the work done during rolling, which is consistent with the findings of this study.

*K*' and *K*" values were positively correlated with work done. These parameters indicate the consistency of the dough under oscillatory shear; higher consistency requires more energy to shape the dough between the rollers.

Creep compliance parameters showed strong negative correlations with work done during rolling. Compliance refers to the amount of material deformation under constant applied stress. The negative correlation between work done and compliance indicates that samples with higher compliance experienced greater deformation under the same stress, while the rolling process had the opposite effect. Another parameter obtained in the creep test is zero shear viscosity, which indicates the molecular interaction of the matrix with flow. There was a strong positive correlation between *µ*_0,c_ and work done, indicating that higher *µ*_0,c_ corresponds to a lower tendency to flow, which requires more energy to induce flow.

In the recovery compliance parameters, the first and second Kelvin-Voigt element compliance values were negatively correlated with work done during rolling, for reasons similar to those observed in creep compliance, since the recovery mechanism of the network was parallel to the creep process. Another similarity to the creep compliance is *µ*_0,r_ value, which was positively correlated with work done. A shorter retardation time of recovery phase was also positively correlated with work done during rolling.

For the extensional rheological parameter, *K*_B_ was found to be positively correlated with work done during rolling. This is because the main deformation mechanism during the rolling process is attributed to the extensional properties of the samples (*4*, *5*). The work value was calculated from the energy requirement of the rolling process, which is characterised by the extensional flow. Extensional stress was directly proportional to *K*_B_ and work done; therefore, these values were well correlated.

### Microstructure of dough samples

**Fig. 6:**
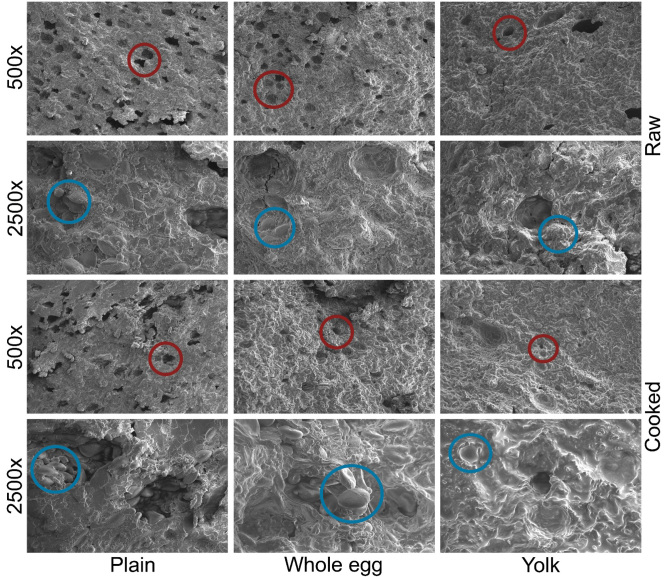
Microstructure of the dough samples before and after cooking. Red circles indicate air bubbles and blue circles indicate starch granules

The microstructure of the dough samples obtained from SEM analysis before cooking is shown in Fig. 6. In the plain sample, air bubbles are evenly distributed throughout the matrix. As the magnification value increases, smaller starch granules can be easily seen. Similarly, Alireza Sadeghi and Bhagya (*28*) studied pasta dough enriched with varying amounts of mustard protein isolate and found comparable results. They attributed the small holes and cracks to insufficient gluten network formation during kneading. They pointed out that the addition of mustard protein isolate created a matrix surrounding the starch granules. Unlike the plain and whole egg samples the yolk sample exhibited a smoother texture due to the coating of the starch granules by the lipid content of the egg yolk. Herawati *et al.* (*29*) studied the impact of different egg fractions on gluten-free noodles and found that the egg yolk provided a more intact structure around the starch granules, which aligns with this study. Fig. 6 shows that all samples contain air bubbles. In industrial production, this issue is mitigated by kneading the dough under vacuum conditions.

After cooking, a significant change was observed in the size of the starch granules formed by gelatinisation. In the plain sample, the the bubbles were partially filled with the starch granules. In addition to the starch granules, the egg protein formed a network between the granules, as seen in the whole egg sample. The changes observed after cooking were similar to those described by Rajeswari *et al.* (*30*), where the yolk sample produced a smoother surface on the starch granules. In the study by Hager *et al.* (*31*), the effect of cooking on pasta was explained by matrix enhancement due to protein coagulation around the starch granules, resulting in stabilisation of the dough, which was consistent with the findings for the whole egg sample.

## CONCLUSIONS

The frequency sweep test conducted in the linear viscoelastic region characterised the viscoelasticity of the model pasta dough samples. Power law model constants showed that the consistency of the samples was different, and the yolk sample was found to have the highest consistency. The creep recovery test revealed the compliance behaviour of the dough samples, which could be an important process parameter for dough rolling and shaping operations. The samples displayed different strain responses, attributable to the gluten network in the matrix. Large amplitude oscillatory shear tests showed that all samples exhibited intracycle strain stiffening and shear thinning behaviour at higher strain values. The area enclosed by the Lissajous-Bowditch curves was the greatest in the yolk sample because of the higher energy required to break down the network. Extensional rheology revealed that the samples obeyed power law during extension, and the behaviour was strain thinning within the measurement range of the extensional rheology test.

In the microstructure analysis of the model pasta dough, the samples were analysed before and after cooking. Holes were more frequent in the plain and whole egg samples than in the yolk sample, where a lipid layer formed over the surface of the starch granules. After cooking, the starch granules were swollen, and protein network formation was observed in the samples containing the whole egg or egg yolk.

The dough-rolling process demonstrated that the energy required to roll out model pasta dough is closely linked to its rheological properties. This study aims to provide insights into the behaviour of real-world pasta dough based on the data obtained from the model pasta dough. The findings indicate that the rheological properties of the dough can be adjusted by selecting different ingredients, which will optimise the pasta dough rolling process. Although this research was conducted on a model dough system designed to simulate the rolling process, these adjustments will enable more accurate process parameters for actual pasta production.

Overall, this work provides a novel and integrated rheological framework for understanding the mechanical behaviour of model pasta dough with the addition of whole egg or egg yolk. By correlating various rheological techniques with rolling energy, this study offers new insight into how the addition of whole egg or egg yolk affects dough consistency, network integrity, and processing energy requirements. These findings are particularly relevant for designing ingredient formulations and processing strategies aimed at improving the efficiency and quality of pasta production.
